# Mapping of the GEVA Items to the ICF: Preliminary Results Based on the Content of a Tool Guide Used to Assess the Needs of Persons With Disabilities in France

**DOI:** 10.3389/fresc.2021.721685

**Published:** 2021-09-14

**Authors:** Marie Cuenot

**Affiliations:** École des Hautes Etudes en Santé Publique, School of Public Health, Rennes, France

**Keywords:** ICF, assessment, mapping, France, GEVA disability assessment tool

## Abstract

The aim of this research is two-fold. Firstly, mapping the GEVA items on to the ICF will allow identifying those items that are covered by the ICF and assist in improving the data collection process. Secondly this work will provide a first exploration of the items that are not covered by the ICF and that could lead to potential proposals for updating the ICF. The preliminary results show that the items of the GEVA 2008 general version are partly covered by the ICF 2017 Browser version categories. In every section of the GEVA, some of the items might be coded with ICF codes coming from the following ICF components: Body functions, Activities and Participation, Environmental factors, Personal factors. The items of the section 6 remains those mostly covered by the ICF. Throughout the GEVA, many environmental factors are documented. Although further analysis is needed to better inform the use of qualifiers (performance, capacity, satisfaction) together with the activities and the environmental factors, the identified ICF codes could assist in improving the data collection process. Finally, some items might be discussed to become potential ICF updates proposals.

## Introduction

How should the needs of persons with disabilities be assessed so as to enable to live with optimum independence, and the means to live and participate in accordance to their wishes? In the last 50 years, France has developed a succession of laws progressively building a legal framework that aims to ensure the rights to individualized compensation measures and the rights for general accessibility to all.

The main concepts, principles, values, and rights are well-known and accepted. However, their implementation may be considered as an on-going search for improvements.

France has been involved in several international processes such as (1) the work related to the concept of disability. This includes the collaboration with WHO during the revision of the International Classification of Impairments, Disabilities, and Handicaps (1980), and the on-going maintenance of the International Classification of Functioning, Disability, and Health (ICF, WHO, 2001) and (2) the work related to the UN Convention on the rights of persons with disabilities (2006) development process. In 2010, France, together with the European Union, ratified the UN Convention.

France may broadly be described as a welfare state, and accordingly the provision for persons with disabilities needs is based on the recognition of an administrative status, namely “persons with disabilities.” The administrative system is complex and depends on the origin of the impairments or disabilities (work, health, army, life accident, etc.), and different allowances may be granted.

The 2005-102 Law “For equal rights and opportunities, participation and citizenship of persons with disabilities” has provided a new framework focusing on the right for compensation and accessibility. Firstly, this law gives a definition of disability as: “any limitation to activity or restriction to participation to life in society, that one may undergo in one's environment, due to substantial, long-term or permanent alteration of one or more physical, sensory, mental or cognitive functions, severe multiple and profound disabilities or a disabling health condition.” Although this definition does contain the concepts found in the ICF, as underlined by the UN Special Rapporteur on the rights of persons with disabilities (1): “[…] the definition of disability […] focuses on the impairment, instead of a person's interaction with the environment and existing barriers, and should therefore be revised.” Hence, the implementation of the concepts might be improved.

Nevertheless, this law has led to the set-up of, in each of the 102 French administrative territorial entities, a one-stop counter [Maison Départementale des Personnes Handicapées (MDPH)] that manages the granting of several different disability benefits, one of which being the Prestation de compensation du handicap “PCH” for citizens aged under 60 years. The MDPHs are in charge of assessing the situations and needs for the compensatory support of persons with disabilities. Each is composed of two bodies: (1) a multidisciplinary team (medical doctors, occupational therapists, psychologists, social workers, etc.) in charge of the assessment of the needs; (2) an executive board, the “Commission for the rights and independence of persons with disabilities,” composed of professionals as well as the representatives of organizations of persons with disabilities. These commissions make all decisions related to the provision of financial, technical, and human aid based on the multidisciplinary assessment, related to the development of a personalized compensation plan. The network of local authorities is supported by a national central authority (national fund of solidarity for autonomy—Caisse Nationale de Solidarité pour l'Autonomie (2) in charge of the implementation of a disability policy throughout the country.

In 2008, a national decree (2008-110, February 6, 2008) provided a guide “Guide d'évaluation multidimensionnelle” (GEVA), for the multidimensional assessment of the needs of persons with disabilities. The aim is to ensure, to the greatest extent possible, equality in the treatment of requests of individuals, equity in granting benefits, and a harmonized countrywide assessment of the situation of persons with disabilities in drawing up individualized support plans. It allows the gathering of information about the situation of an individual, summarizes the main key points of the assessment, and supports the decision process. Section 6 of GEVA aiming at supporting the decision taken to allocate the PCH has been explicitly built based on ICF categories and qualifiers.

The general version of this guide, 40 pages, has been developed in 2008 (2) and was first designed to assess the needs of adults aged under 60 years. Several complementary versions do exist, especially some focusing on education and children. As mentioned in a 2014 national study, “Generally speaking, the GEVA is used as a reference framework, but many MDPH have adapted it making their own and simplified versions. Professionals involved in assessment use the sections that seem most relevant and useful to them in relation to the situation. […] A majority of the MDPH use a tool for gathering information at the individual's home. Half of these tools are *ad-hoc* tools that contain GEVA items” [(3), p. 36, Translation M. Cuenot].

This initial version provided to the professionals contains no codes and no formal procedure designed to be used for data collection. Only in the annex of the 2008 decree, some hierarchical *ad hoc* codes are provided for each item and each possible response. The hierarchy is organized by the order of GEVA sections.

The aim of this research is two-fold. Firstly, mapping of the GEVA items onto the ICF will allow to identify those items that are covered by the ICF and assist in improving the data collection process. Secondly, this work will provide the first exploration of the items that are not covered by the ICF, and then could lead to potential additional proposals for updating the ICF. After introducing the method used for this mapping, some preliminary results will be described and then discussed.

## Methods

The items of the general 2008 paper-based version of the GEVA have been used as a reference version for this mapping (2). They have been translated into English by the author of this paper. Table 1 gives an overview of the content divided up into eight sections. Our analysis did not only take into account the GEVA Section 6 explicitly related to Activities, but also the GEVA Sections 1–5, the Sections 7 and 8 being merely the summaries of the other sections. We hypothesized that some other sections could also be coded with the ICF.

**Table 1:** Overview of the GEVA 2008 sections and corresponding covered ICF components.

**Sections**	**Overview of content**	**ICF components**
**Identification:**	Title; Name; ID; Initial reasons of assessment; Life project and wishes of the person; Acquaintances' or legal representative's opinion	PF
**Section 1:** Family, social, and financial situation	Family status; List of persons currently in the household Social situation in relation to education or employment, including child care arrangements for young children Individual financial resources Elective offices; related needs for human assistance.	PF; D; E
**Section 2:** Housing and living conditions	Housing: accommodation, housing types and characteristics (accessibility; comfort) Services and building accessibility Transportation: school; human assistance and adaptations	PF; D; E
**Section 3:** Training and professional path	Education path: background and orientation; learning assesment and adaptations; professionals' opinion Professional path: plan; situation; status; workstation and working conditions arrangements; job coach or psychologist's opinion; arrangements for maintaining a job; skills assessment.	PF; D; E
**Section 4:** Medical information	Pathological origin of the disabilities; medical background; clinical information (vision; hearing); symptoms; foreseeable evolution Impairments (see regulatory framework); healthcare provision Constraints: personal assistive devices; food; natural environment	(ICD-10)B; PF; D; E
**Section 5:** Psychological assessment	(Neuro-)Psychological and/or memory assessment	PF
**Section 6:** Activities	1. General tasks and demands, and interpersonal relationships 2. Mobility 3. Self-care 4. Communication 5. Domestic and daily life 6. Applying knowledge and learning 7. Tasks and demands related to education 8. Tasks and demands related to employment	B; D; E
**Section 7:** Assistance and care provided	Human assistance (acquaintances, professionnals, joint collaboration) Organization of a usual day and week Professionals related to the education project Summary-Key points to be emphasized Discussion with the concerned person about the assessment Eligibility criteria (severe and complete difficulties identified) Identified needs in relation to different activities	PF; B; D; E
**Section 8:** Assessment summary	Summary of assessment, intended to the Commission's attention	PF; B; D; E

The linking rules formulated by Cieza et al. (4, 5) have guided the mapping work. The ICF 2017 browser version, currently available online, has been used as the reference ICF version for this work. In a general statistic approach, every different GEVA item has been counted in Sections 1–6, thus not including duplicated ones and counting only one ICF category for one GEVA item. The main results presented here are based on a qualitative analysis of the content of the GEVA for the aim is rather showing which ICF categories may be used to code the GEVA items than showing how many GEVA items are coded (several GEVA items may correspond to one same ICF category or are duplicated several times throughout the GEVA).

## Results

Preliminary results show that the GEVA items are partly covered by the ICF. The general statistic approach allows to estimate that 80% (343 of 435) of the GEVA items can be covered by the ICF categories. In each of the six considered sections of the GEVA, some of the items may be coded with the ICF codes. Hence, not only the items of the Section 6 of the GEVA explicitly relate to Activities but also some items of the other GEVA Sections 1–5 belong to the universe of the ICF. The covered items are related to the following ICF components: Body functions (B), Activities and Participation (D), Environmental factors (E), and Personal factors (PF).

Some items are covered by other international classifications. In Section 4, the information related to diseases and rare diseases and to hearing and vision could be coded with ICD-10 codes. Some items may also be covered by the 1980 ICIDH categories. Indeed, some parts of the national regulatory framework still use impairments and disabilities to specifically fix the disability rates (Guide-barème). These impairment-related items may be mapped onto some ICF B categories and a related qualifier (Table 2).

**Table 2:** GEVA items covered by ICF categories (exact matches).

**GEVA item**	**ICF 2nd or 3rd-level codes**
**Section 4**	**B**
Intellectual and cognitive impairments	b1 Mental functions + qualifiers
Mental impairments	b1 Mental functions + qualifiers
Hearing impairments	b230 Hearing functions + qualifiers
Language impairments	b3 Voice and speech functions + qualifiers
Vision impairments	b210 Seeing functions + qualifiers
Visceral impairments	Functions related to the digestive system (b510-b539) + qualifiers
Motor impairments	b7 Neuromusculoskeletal and movement-related functions + qualifiers
**Section 6**	**B**
1.1 Orientation to time	b1140 Orientation to time
1.2 Orientation to space	b1144 Orientation to space
1.4 Memorize	b144 Memory functions
4.2.1 Hearing sounds	b2300 Sound detection
4.2.5 Localization of sound source	b2302 Localization of sound source
4.3 Seeing	b210 Seeing functions
8.8.1 Seeing colors	b21021 Color vision
8.8.2 Depth perception	b21000 Binocular acuity of distant vision
3.6.1 Using one's respiratory functions	b440 Respiration functions
2.15 Two-hand coordination	b7602 Coordination of voluntary movements
**Section 2**	d850 Remunerative employment
**Section 6**	**D**
1.3 Focusing attention	d160 Focusing attention
1.5 Making decisions	d177 Making decisions
1.6 Taking initiatives	d177 Making decisions
1.6.2 Spontaneously forming relationships	d7200 Forming relationships
1.6.3 Spontaneously undertaking a simple activity	d2100 Undertaking a simple task
1.6.4 Spontaneously undertaking a complex activity	d2101 Undertaking a complex task
1.7 Managing one's own safety 1.7.1 Not endangering other's life or one's own life	d570 Looking after one's health
1.7.2 Adapted reaction in a hazardous situation	d240 Handling stress and other psychological demands
1.8 Respecting social rules 1.9 Relating to others according to social rules	d7203 Interacting according to social rules
1.10 Control one's behavior in interactions with others	d7202 Regulating behaviors within interactions
1.11 Relating with peers	d7402 Relating with equals
1.12 Having intimate relationships	d770 Intimate relationships
2.1 Standing	d4104 Standing
2.1.1 Lying down	d4100 Lying down
2.1.2 Sitting	d4103 Sitting
2.2 Transferring oneself	d420 Transferring oneself
2.3 Shifting the point of support	d4106 Shifting the body's center of gravity
2.4 Maintaining a sitting position	d4153 Maintaining a sitting position
2.5 Maintaining a standing position	d4154 Maintaining a standing position
2.6 Walking	d450 Walking
2.7.1 Moving around within the home	d4600 Moving around within the home
2.7.2 Moving around outside the home	d4602 Moving around outside the home and other buildings
2.8 Climbing stairs	d4551 Climbing
2.9 Using public transportation	d4702 Using public motorized transportation
2.10 Using one's own vehicle	d4701 Using private motorized transportation
2.11 Driving a vehicle	d475 Driving
2.14 Fine hand use	d440 Fine hand use
2.16 Lifting and carrying objects (including while moving around)	d430 Lifting and carrying objects
3.1 Washing oneself	d510 Washing oneself
3.2 Caring for body	d520 Caring for body parts
3.3 Toileting and using the toilets	d530 Toileting
3.4 Putting on and taking off clothes	d5400 Putting on clothes; d5401 Taking off clothes
3.5 Eating, drinking	d550 Eating; d560 Drinking
3.6 Looking after one's health	d570 Looking after one's health
3.6.2 Caring for oneself	d5702 Maintaining one's health
3.6.4 Monitoring one's own diet	d5701 Managing diet and fitness
4.1 Speaking	d330 Speaking
4.2.2 /0.3 /0.4 Understanding others' speaking face to face/in a group/in a noisy environment	d310 Communicating with—receiving—spoken messages
4.4 Using communication devices and techniques	d360 Using communication devices and techniques
4.4.1 Using a phone	d3600 Using telecommunication devices
4.4.2 Using other communication devices and techniques	d3609 Using communication devices and techniques, unspecified
4.5 Understanding a simple sentence	d3100 Communicating with - receiving - simple spoken messages
4.6 Maintaining a conversation	d350 Conversation
4.7 Producing and receiving non-verbal messages	d315 Communicating with - receiving - nonverbal messages; d335 Producing non-verbal messages
5.1 Shopping	d6200 Shopping
5.2 Preparing a simple meal	d6300 Preparing simple meals
5.3 Doing housework	d640 Doing housework
5.4 Taking care of clothes and garments	d6400 Washing and drying clothes and garments
5.5 Taking care of one's own family	d660 Assisting others
5.6 / 5.6.1 Managing one's own money daily and administrative tasks	d860 Basic economic transactions (managing one's budget, administrative tasks)
5.6.2 Managing one's bank account	d865 Complex economic transactions (managing one's bank account)
5.8 Having informal relationships with neighbors	d7501 Informal relationships with neighbors
5.9 Participating in community, social and civic life	d9 Community, social and civic life
5.9.3 Participating in local life	d950 Political life and citizenship
6.1 Reading	d166 Reading
6.2 Writing	d170 Writing
6.3 Calculating	d172 Calculating
6.4 Acquiring know-how skills; 6.5 Applying know-how skills	d155 Acquiring skills
7.1 Learning to read	d140 Learning to read
7.2 Learning to write	d145 Learning to write
7.3 Learning to calculate	d150 Learning to calculate
7.6 / 8.1/ 7.6.9 Respecting basic rules in relation to education/employment; Respecting school rules	d7203 Interacting according to social rules
7.6.8 / 8.6 Working with a team, in relation to education/employment	d7402 Relating with equals
8.1.3 Respecting hierarchical relations	d8451 Maintaining a job
8.1.4 Participating in meetings	d3551 Discussion with many people
8.7 / 8.7.1 Physical tasks/Lifting and moving heavy objects	d430 Lifting and carrying objects
8.7.2 Trunk twisting while working	d4305 Putting down objects
8.7.3 Working in various positions	d4309 Lifting and carrying, unspecified
8.7.4 Working in a squatting position	d4151 Maintaining a squatting position
8.7.9 Using one's feet to use a machine	d4350 Pushing with lower extremities

The section in which the GEVA items are mostly covered by the ICF D chapters remains Section 6 (Table 2). Each item of this section is explicitly intended to be assessed through the two qualifiers: functional capacity and performance. Functional capacity should be assessed with the following scale, which is similar to the one recommended for the ICF: “0”: No difficulty; “1”: Mild difficulty; “2”: Moderate difficulty; “3”: Severe difficulty; “4”: Total difficulty; and “9”: Non-applicable. Performance should be assessed with another scale: a-activity performed alone; b-activity partially performed with human assistance; c-activity performed with continued assistance; and d-activity not performed.

This performance scale provides the information about the environment, especially mentioning the potential impact of human assistance on the performed activity. This means that conceptually the data that might be collected at that stage should contain one related to D and one related to E. The following general five types of E are required to be documented as facilitators or barriers during the performance assessment: human environment [ICF chapter e3 Support and relationships]; technical assistance [e1 Products and technology] housing adaptations [e1158 Products and technology for personal use in daily living, other specified]; services [e5 Services, systems, and policies]; and animal assistance [e350 Domesticated animals]. Additionally, in this GEVA Section 6, a list of items are given for a more in-depth assessment of the human, technical, and animal assistance provided in each life domain. A related satisfaction assessment scale is eventually included to collect the point of view of the individual about the situation: complete satisfaction; satisfaction; mild satisfaction; and no satisfaction.

Hence, throughout the GEVA sections, many E are listed that may be documented. Table 3 presents the corresponding ICF E categories that are covered. Some require additional information to know whether they have an impact on the individual situation, as a facilitator or an obstacle. Others only require to be identified as being provided or not. A more in-depth analysis would allow more information on how they are formulated in relation to the ICF E qualifiers. Nevertheless, these preliminary results show that the ICF E Chapters 1, 2, 3, and 5 are documented throughout the GEVA items. The categories of Chapter 4 related to Attitudes are not represented as such.

**Table 3:** ICF Environmental factors documented throughout the GEVA and related GEVA sections.

E1 Products and technology	6: General Environmental factor technical environment
e1101 Drugs	4: Regular treatments
e1108 Products or substances for personal consumption, other specified	4: Food constraints
e1151 Assistive products and technology for personal use in daily living	4: Urinary ostomy and catheter, digestive ostomy, gastrostomy, jejunostomy, tracheotomy, ventilation unit
e1158 Products and technology for personal use in daily living, other specified	4: Dependence on another machine 6: General Environmental factor housing
e120 Products and technology for personal indoor and outdoor mobility and transportation	2: Adapted vehicule
e1201 Assistive products and technology for personal indoor and outdoor mobility and transportation	4: Walking sticks, wheelchair
e1251 Assistive products and technology for communication	4: Hearing aid
e130 Products and technology for education	3: Adaptations during school assessments
e1301 Assistive products and technology for education	6: Adapted teaching material, computers
e135 Products and technology for employment	3: Workstation accommodation
e150 Design, construction and building products and technology of buildings for public use;	2: Accessibility of postal, and education facilities and services
e1500 Design, construction and building products and technology for entering and exiting buildings for public use	2: Accessibility of the housing
e1501 Design, construction and building products and technology for gaining access to facilities inside buildings for public use	2: Lift in the housing
e1508 Design, construction and building products and technology of buildings for public use, other specified	2: Other elements of accessibility of the housing
e1550 Design, construction and building products and technology for entering and exiting of buildings for private use	2: Accessibility of shops, banks
e1551 Design, construction and building products and technology for gaining access to facilities in buildings for private use	2: Accessibility of living room, kitchen, bedroom, toilets, bathroom, vertical and horizontal inside circulation
e1650 Assets	2: Owner of housing
e198 Products and technology, other specified	4: Equipment
e225 Climate	4: Weather
e2250 Temperature	4: Cold, heat
e2251 Humidity	4: Humidity
e240 Light	4: Light
e2450 Day/night cycles	6: Night work
e2458 Time-related changes, other specified	6: Working assuming schedules modifications
e250 Sound	4: Noise
e2500 Sound intensity	6: Working in a noisy environment
e255 Vibration	6: Working in an environment with vibrations
e260 Air quality	6: Working in specific respiratory environments
e2601 Outdoor air quality	4: Dust, chemical agents
e298 Natural environment and human-made changes to environment, other specified	4: Other external factor constraints
e3 Support and Relationships	6: General Environmental factor human environment
e310 Immediate family	6: Spouse, child, parent, brother/sister, other parent
e325 Acquaintances, peers, colleagues, neighbors and community members	6: Friend, neighbor, mutual support group
e340 Personal care providers and personal assistants	3: Human assistance during school assessments 4: Personal care assistant 6: Personal assistant at school
e350 Domesticated animals	6: General Environmental factor animal environment
e355 Health professionals	4: PT, OT, nurse, speech therapist, psychologist
e398 Support and relationships, other specified	6: Cued speech interpreters, technicians in writing, sign language interpreters
e399 Support and relationships, unspecified	6: Associated to the general performance qualifier levels b and c
e5 Services, systems and policies	6: General Environmental factor services
e5250 Housing services	6: Types of housing facilities
e5258 Housing services, systems and policies, other specified	6: Different types of medico-social facilities
e5300 Utilities services	2: Heat, gaz, water, electricity, other
e5350 Communication services	2: Accessibility of postal services
e5400 Transportation services	2: Accessibility of transportation facilities
e5500 Legal services	6: Legal protection
e5550 Associations and organizational services	2: Accessibility of cultural and leisure services and facilities
e5650 Economic services	2: Accessibility of bank services
e5652 Economic policies	2: Insurance premiums and restrictions
e5758 General social support services, systems and policies, other specified	6: Different types of medico-social supports
e580 Health services, systems and policies	4: Healthcare and night care provision, meals delivery services 6: Different types of medico-social supports
e5808 Health services, systems and policies, other specified	6: Different types of hospitalization
e585 Education and training services, systems and policies	3: Types of schooling/training/examination facilities and services 6: Remote learning and home schooling services, specialized assistance services for pupils, homework support services
e5850 Education and training services	2: Accessibility of school, pre-school, kindergarden services
e590 Labor and employment services, systems and policies	3: Types of working conditions and workstation arrangements 6: Job seeking and support services
e5908 Labor and employment services, systems and policies, other specified	6: Support and work institutions
e598 Services, systems and policies, other specified	2: Accessibility of other services

Throughout the GEVA, some items are also related to PF. Some additional qualitative information is listed in the GEVA and relates to proxy and points of view of professionals about the situation of an individual (psychological, medical, educational, apprenticeship, and professional).

Finally, some GEVA items might be covered by the ICF but no exact match is really possible. Table 4 presents them together with the potentially lacking concept. They are mainly related to D and E. Other GEVA items cannot at all be coded with the ICF for they require qualitative information, either the point of view of other persons in relation to the main one asking for an assessment, or administrative information about some applications in progress and time-related information. The satisfaction qualifier is also not a part of the ICF.

**Table 4:** GEVA items not completely covered by the ICF.

**GEVA item and related GEVA Section**	**Most precise ICF categories**	**Potential additional concepts related to ICF chapters**
**Section 1**		
Childcare related services	e5850 Education and training services	Child care facilities (e)
- Engaging in political life - Participation in councils where DPO's representatives are appointed as members by law: Local councils; national or European parliament; other	d950 Political life and citizenship	LocalNationalTransnationalOther specified
Human assistance required in relation to political life;Other needs	e340 Personal care providers and personal assistants e5950 Political services	Human assistance related to political engagement (e)
**Section 3–4**		
School assessment criteria (adaptations, additional time)	d820 School education	Learning assessment criteria
Additional time necessary for care provision	e580 Health services, systems and policies	Additional time
Infectious agents	e2201 Animals	Infectious agents (e)
**Section 6**		
1.6.1 Spontaneously asking for help 3.6.3 Expression of a need for healthcare 5.9.2 Expression of a request for help in relation to one's own rights	d570 Looking after one's health d940 Human rights	Asking for help or assistance
2.12 / 13 Grasping using the dominant hand/non-dominant hand	d4401 Grasping	Dominant hand/Non-dominant hand
3.6.5 Managing one's daily rest	d570 Looking after one's health	Managing one's need for some rest
5.9.1 Managing one's free time, participating in cultural or sport activities	d920 Recreation and leisure	Managing one's free time
5.10 Going on holiday	d9208 Recreation and leisure, other specified	Going on holiday
7.4 Learning communication techniques	d3608 Using communication devices and techniques, other specified	Learning how to use communication techniques
7.5 Learning basic social rules	d7208 Complex interpersonal interactions, other specified	Learning basic social rules
7.6.3/8.2 Organizing one's work 7.6.4/8.3 Checking one's work 7.7 Getting organized in the classroom	d820 School education d8451 Maintaining a job	Organizing one's workChecking one's workGetting organized in the classroomIn relation to educationIn relation to employment
7.6.7 Adaptation to school life	d820 School education	Getting used to school life
7.8 Using learning materials 7.9 Using materials adapted to one's disabilities	d820 School education	Using learning materialsUsing materials adapted to one's disabilities
7.10 Taking notes	d820 School education	Taking notes
7.11 Adaptation to examination and assessment conditions	d820 School education	Adaptation to examination and assessment conditions
7.12 Participating in after-school activities	d820 School education	Participating in after-school activities
8.7.5/8.7.6 Working at height (scale, roof)	d4158 Maintaining a body position, other specified	Working at height
8.7.7 Working with the dominant arm above the shoulders' height 8.7.8/non-dominant	d4308 Lifting and carrying, other specified	Working with the dominant/non-dominant arm above the shoulders' height
8.8.5 Using hazardous tools or machines	d2402 Handling crisis	Using hazardous tools or machines
8.8.9 Working in environments with risks for the skin	d2402 Handling crisis	Risks in relation to the skin

## Discussion

These preliminary results of this GEVA-ICF mapping provides a first overview of the ICF coverage potential. The categories stemming from the D chapters and the Environmental chapters, together with the corresponding qualifiers, are well-represented. Not completely covered items deal in particular with education and employment (see Table 4). They raise some issues on how to better implement specific adaptations in these two areas and others so as to facilitate the coding and collection of some precise information.

This also underlines the needs to better document the way of interaction of these components together in some specific areas, in particular such as education or employment, which are the two major areas in the individual life project.

In the ICF education category (d820), additional information could then be added in relation to assessment/examination criteria, organizing and checking one's own work, getting organized in the classroom, getting used to school life, using learning (adapted) materials, taking notes, and participating in after-school activities. Several GEVA items also relate to the learning process: learning how to use communication techniques, or basic social rules, asking for help or assistance.

In the employment area, additional information could be added about organizing and checking one's work, working at height, working with the dominant/non-dominant arm, and working in hazardous environments.

Furthermore, the GEVA satisfaction qualifier is currently used and allows a better understanding of the impact of the E. A more in-depth analysis is needed to firstly better inform the use of qualifiers together with E and secondly to improve the identification of the items directly covered by the ICF definition of PF and others that might not be covered but which provided relevant information for a better understanding of the situation and project of an individual.

In relation to E and PF, there is also a need to distinguish on one hand the content directly inspired by the French regulatory framework and on the other hand the content based on the ICF concepts and implementation rules. The GEVA has been designed in accordance with the French legal framework and not its whole content may be applied to other social and cultural contexts without adaptation.

The GEVA items not completely covered by the ICF might benefit from the discussions with other ICF experts so as to better evaluate them as potential ICF update proposals.

## Conclusion

This mapping approach shows that firstly the items of the GEVA 2008 general version are partly covered by the ICF 2017 Browser version categories. In every section of the GEVA, some of the items might be coded with the ICF codes obtained from the following ICF components: B, D, E, and PF. The items of Section 6 remains those mostly covered by the ICF. Throughout the GEVA, many E factors are documented. Although further analysis is needed to better inform the use of qualifiers (performance, capacity, and satisfaction) together with the D, and the E, the identified ICF codes could assist in improving the data collection process. Finally, some items might be discussed to become potential ICF update proposals.

## Data Availability Statement

The raw data supporting the conclusions of this article will be made available by the authors, without undue reservation.

## Author Contributions

The author confirms being the sole contributor of this work and has approved it for publication.

## Conflict of Interest

The author declares that the research was conducted in the absence of any commercial or financial relationships that could be construed as a potential conflict of interest.

## Publisher's Note

All claims expressed in this article are solely those of the authors and do not necessarily represent those of their affiliated organizations, or those of the publisher, the editors and the reviewers. Any product that may be evaluated in this article, or claim that may be made by its manufacturer, is not guaranteed or endorsed by the publisher.

## References

[1] Human Rights Council. Visit to France. Report of the Special Rapporteur on the Rights of Persons With Disabilities. United Nations General Assembly (2019).

[2] Caisse nationale de solidarité pour l'autonomie (CNSA). GEVA – Guide d'évaluation multidimensionnelle. (2008). Available online at: https://www.cnsa.fr/documentation/geva_graphique-080529-2.pdf

[3] Ministère des affaires sociales et de la santé Haut conseil de la santé publique Cekoïa Conseil. Enquête quantitative sur les modes d'évaluation et de traitement des demandes de compensation du handicap par les MDPH, Rapport final. (2014). Available online at: https://www.google.com/url?sa=t&rct=j&q=&esrc=s&source=web&cd=&ved=2ahUKEwi1oMHi46rwAhXHDGMBHZTfA2wQFjABegQIBhAD&url=https%3A%2F%2Fwww.hcsp.fr%2FExplore.cgi%2FTelecharger%3FNomFichier%3Dhcspr20150511_evalbesoinhandicapmdph.pdf&usg=AOvVaw2WklIiwguHt7e3benEF0x9

[4] Cieza A Fayed N Bickenbach J Prodinger B. Refinements of the ICF linking rules to strengthen their potential for establishing comparability of health information. Disabil Rehabil. (2019) 41:574–83. 10.3109/09638288.2016.1145258 26984720

[5] Cieza A Geyh S Chatterji S Kostanjsek N Üstün B Stucki G. ICF linking rules: an update based on lessons learned. J Rehabil Med. (2005) 37:212–8. 10.1080/16501970510040263 16024476

